# Massive 4-Gland Parathyroid Hyperplasia Initially Detected as a Parathyroid Adenoma

**DOI:** 10.1210/jcemcr/luad173

**Published:** 2024-01-05

**Authors:** Sophie Nicolich-Henkin, Michael B Goldstein, Emma Roellke, John P Bilezikian, Gary D Rothberger

**Affiliations:** Division of Endocrinology, NYU Long Island School of Medicine, Mineola, NY 11501, USA; Division of Endocrinology, NYU Long Island School of Medicine, Mineola, NY 11501, USA; Department of Medicine, NYU Long Island School of Medicine, Mineola, NY 11501, USA; Department of Medicine, Division of Endocrinology, Vagelos College of Physicians and Surgeons, Columbia University, New York, NY 10032, USA; Division of Endocrinology, NYU Long Island School of Medicine, Mineola, NY 11501, USA

**Keywords:** primary hyperparathyroidism, hyperplasia, adenoma, sestamibi, ultrasound

## Abstract

Abstract: Parathyroid adenoma (PA) and parathyroid hyperplasia (PH) are common causes of primary hyperparathyroidism (PHPT), for which the only definitive treatment is surgery. Abnormalities in the parathyroid glands can be identified with various imaging modalities including ultrasound (US), sestamibi scan (MIBI), 4-dimensional computed tomography (4D-CT), and positron emission tomography/computed tomography (PET/CT). While it is not uncommon for parathyroid pathology to be undetected on imaging, this is more typical of low-volume hyperplasia and smaller-sized adenomas. We present the case of a 65-year-old man with PHPT who initially had a solitary parathyroid mass detected by US, but who was ultimately discovered to have massive PH with hyperplastic glands not visualized on US or MIBI. This atypical presentation may help guide providers in decisions on ordering and interpreting various imaging modalities for patients with PHPT. In this case, 4D-CT was the only modality in which large hyperplastic glands were identified, suggesting superior sensitivity. This case also highlights the importance of intraoperative parathyroid hormone testing to aid in diagnostic prediction.

## Introduction

Primary hyperparathyroidism (PHPT) most commonly presents with asymptomatic hypercalcemia discovered on routine screening. It is characterized by findings of hypercalcemia with elevated or inappropriately normal parathyroid hormone (PTH) levels. Parathyroid adenoma (PA) and parathyroid hyperplasia (PH) are common causes of PHPT, with solitary adenomas comprising the majority of cases (∼80%-85%) and multiple gland hyperplasia in a minority of cases (∼15%-20%) [1]. Numerous imaging modalities can be utilized to visualize the parathyroid glands including ultrasound (US), sestamibi scan (MIBI), 4-dimensional computed tomography (4D-CT), and positron emission tomography/computer tomography (PET/CT). While PA and PH are typically not distinguishable on clinical grounds, they may have distinct imaging characteristics [2].

We present the clinical presentation, diagnostic assessment, management, and follow-up of a patient whose initial diagnosis was consistent with a solitary PA. However, he had persistently elevated PTH and calcium levels after surgical removal of a single large right parathyroid mass. No further abnormalities were detected on repeat US, MIBI, or PET/CT. Eventually, multiglandular involvement was detected by 4D-CT with subsequent surgical removal during a re-operative procedure.

## Case Presentation

**Figure 1.**
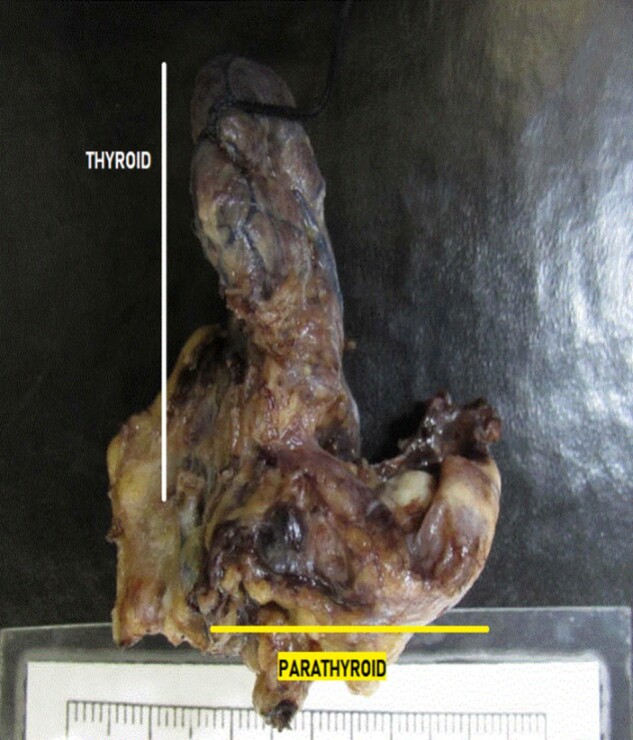
Gross surgical pathology of resected parathyroid gland weighing 500 mg and measuring 2.5 cm in greater length.

A 65-year-old man without family history of parathyroid or calcium disorders was referred for parathyroidectomy for the management of longstanding elevated calcium and PTH levels. The patient reported knowledge of these elevated levels for 15 years; however, he had previously declined further evaluation. He had no prior fractures and no known history of gastrointestinal, renal, or nutritional disorders. He was not on any medications that could predispose to parathyroid hyperplasia. The PTH level was 581 pg/mL (581 ng/L) (reference range, 12-65 pg/mL [12-65 ng/L]) with a concomitant serum calcium of 11.8 mg/dL (2.94 mmol/L) (reference range, 8.6-10.5 mg/dL [2.15-2.62 mmol/L]). Dual-energy x-ray absorptiometry (DXA) scan revealed a T-score of −1.6 at the spine, −2.6 at the left femoral neck, and −5.4 at the left forearm (reference range, ≥ −1.0). Preoperative localization US revealed a solitary well-defined enlarged PA measuring 2.3 × 2.2 × 2.5 cm posterior to the lower pole of the right thyroid lobe, with no other abnormalities appreciated on that study. With one large gland identified, it was presumed that the elevated PTH was due to this solitary adenoma, and the patient was scheduled for surgery. Due to a reported allergic reaction to intravenous contrast agents, 4D-CT was not performed. The patient underwent right inferior parathyroidectomy and right thyroid lobectomy; during the procedure, the patient was found to have a large right inferior parathyroid gland with a cystic component densely adhered to the strap muscles and the right lobe of the thyroid concerning for malignancy, therefore both were removed (Fig. 1). Pathology was initially suggestive of a PA weighing 500 mg and measuring 1.4 × 0.6 × 0.5 cm. The final weight and size may have been smaller on pathology than on initial imaging due to the cystic component of the removed gland. Intraoperative PTH was not measured, but PTH measured 1 day postoperatively was 550 pg/mL (550 ng/L), and steadily increased to a peak of 1890 pg/mL (1890 ng/L) over the following 3 months with associated hypercalcemia ranging from 11.3-14.2 mg/dL (5.65-7.1 mmol/L) over the same time period.

## Diagnostic Assessment

**Figure 2.**
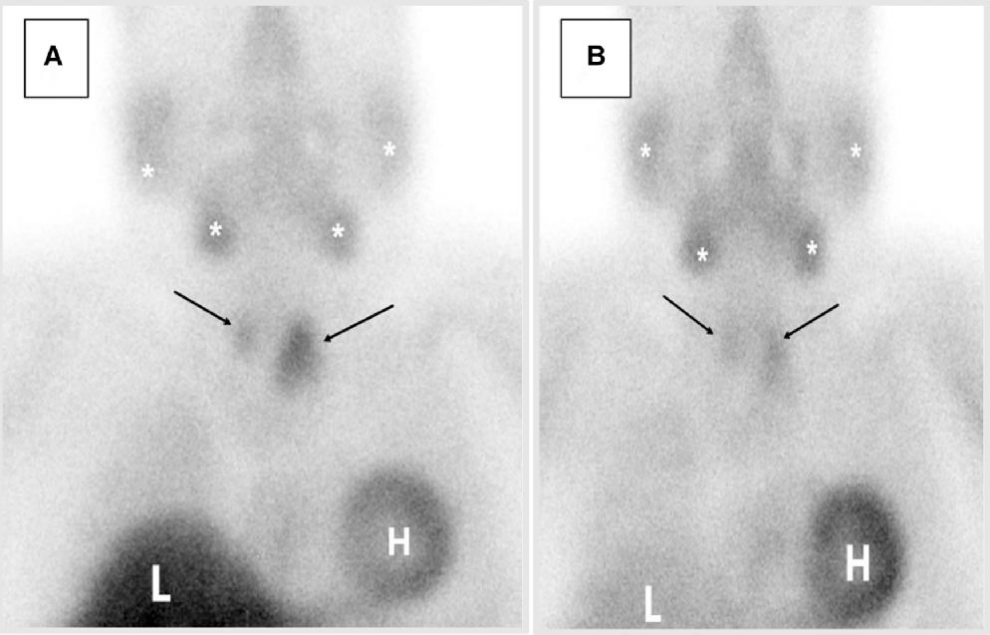
Anterior planar images of the neck and chest acquired (A) 20 minutes and (B) 120 minutes after IV administration of 30.1 mCi 99mTc-sestamibi showed physiological uptake throughout the thyroid gland (black arrows) without discrete focal abnormality to suggest residual parathyroid lesions. Also note, physiological uptake in the liver (L), heart (H) and salivary glands (*).

**Figure 3.**
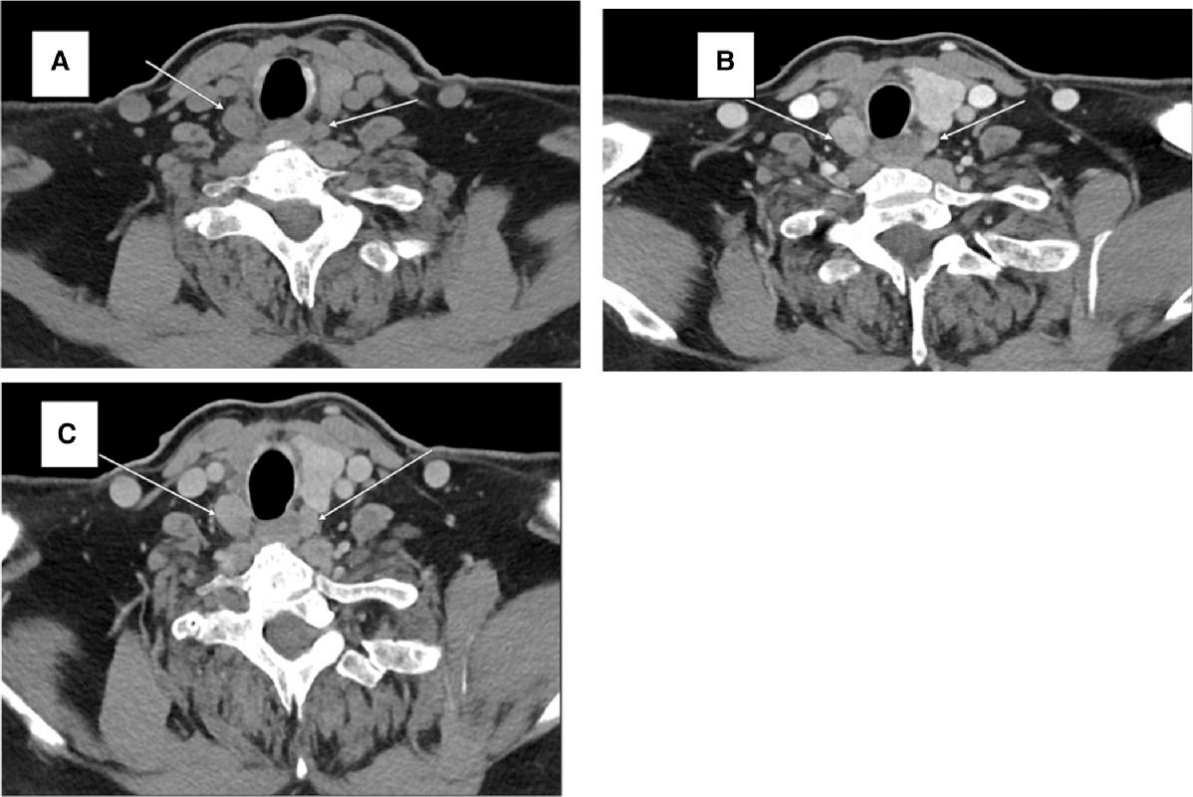
(A) Non-contrast-enhanced axial CT image and (B) IV contrast-enhanced axial CT images during the arterial (20 seconds) and (C) venous (120 seconds) phases of enhancement demonstrated a 1.8 × 1.4 × 3.0 cm and a 1.2 × 0.8 × 2.1 cm (AP × TRV × SAG) hypo-dense lesion with homogenous arterial phase enhancement and delayed phase washout at the level of the cricoid cartridge undersurface, consistent with parathyroid adenomas (white arrows). Additionally, a 0.3 × 0.4 × 0.4 cm parathyroid adenoma was identified posterior to the right carotid artery at the level of the superior aspect of the cricoid cartridge (not shown).

Further imaging was implemented to identify the source of persistent PTH secretion postoperatively. MIBI did not display any definite parathyroid adenoma or ectopic parathyroid tissue (Fig. 2), fluorodeoxyglucose (FDG)-PET/CT did not demonstrate evidence of metabolically active residual or ectopic parathyroid adenoma or metastatic disease, and repeat US did not reveal evidence of parathyroid abnormalities. Ultimately, the patient was pretreated with glucocorticoids and had a 4D-CT that detected multiglandular disease with a 1.8 × 1.4 × 3.0 cm right superior parathyroid gland weighing approximately 3931 mg, 1.2 × 0.8 × 2.1 cm left superior parathyroid gland weighing approximately 1048 mg, and 0.3 × 0.4 × 0.4 cm left inferior parathyroid weighing approximately 25 mg (Fig. 3A-3C).

## Treatment

The patient underwent re-exploration parathyroidectomy with removal of the right superior hyperplastic parathyroid gland weighing 3105 mg and left superior hyperplastic parathyroid gland weighing 1100 mg. The remaining left inferior gland appeared hyperplastic intraoperatively and was estimated to weigh 700 mg. A biopsy was obtained, yielding 40 mg of normal parathyroid tissue, which stained positive for parafibromin. Approximately two-thirds of the gland was then removed and implanted into the forearm.

## Outcome and Follow-Up

One week postoperatively, the PTH level was 5 pg/mL (5 ng/L) with a concomitant serum calcium of 8.9 mg/dL (2.22 mmol/L). Approximately 1 year postoperatively, PTH and serum calcium were normal. The nongrafted arm PTH level was 82 pg/mL (82 ng/L) and the grafted arm PTH level was 294 pg/mL (294 ng/L), indicating intact graft function (reference range, 24-86 pg/mL (24-86 ng/L)). Repeat DXA 18 months postoperatively revealed generalized improvements in bone mineralization with T-scores of 0 at the spine (19.6% improvement), −1.8 at the left femoral neck (11.5% improvement), and −5.1 at the left forearm (3% improvement). The patient declined genetic testing.

## Discussion

PHPT most commonly presents with asymptomatic hypercalcemia. While some patients can be managed with frequent monitoring of serum calcium, renal function, and skeletal imaging, the only definitive treatment is surgery. Prior to the advent of minimally invasive surgical techniques, surgery was performed by bilateral neck exploration, identification of the 4 parathyroid glands with removal of those that appear grossly enlarged, and biopsy of the remaining glands. If all 4 glands were affected, 3.5 glands were removed—a strategy with a success rate of greater than 95% [1]. Today, however, imaging of the parathyroid gland is commonly performed prior to surgery to localize the adenoma for removal and to minimize the need for extensive surgical exploration. Ultrasound, with a sensitivity of 55% to 83.5%, allows anatomic visualization of structural neck pathology without radiation exposure, but it is not able to identify function and has limited ability to detect ectopic parathyroid tissue [2‐4]. MIBI, with a sensitivity of 50% to 85%, can identify hyperfunctioning parathyroid glands by their increased uptake of radiotracer [2‐4]. PET/CT, with a sensitivity of 92% for detection of PA and 68% for PH [5], uses choline-based radiotracers to detect cells with high proliferation rates. 4D-CT, with a sensitivity of 88%, has the benefits of a 3-dimensional CT scan, which allows for imaging of neck and mediastinum, with the added dimension of changes in perfusion over time. This facilitates improved detail in multiplanar images and visualization of differences in perfusion of hyperfunctioning parathyroid glands, thereby providing information about both anatomic location and function [2].

Previous studies comparing PH with PA have not identified significant differences in age, gender, and clinical presentation between these conditions [6, 7], but PA typically presents with higher serum preoperative calcium levels than PH [6, 7]. PH with both familial and sporadic mutations has been documented to have marked heterogeneity in gland size. Notably, patients with PH are also more likely to have lower weight of their largest gland than those with PA [6]. In PH, it is not uncommon for the US and the MIBI to be negative [8]. Patients with PH are more likely to have a negative MIBI compared to those with PA (92% vs 18%), as well as more likely to have a negative US (96% vs 12%) [6], possibly due to the typically lower gland size of PH compared to PA. A study reviewing histological pathology of PA in patients who underwent MIBI found that, in general, larger weight and volume were correlated with positive MIBI. However, even rather large adenomas, as big as 2300 mg have been missed by MIBI [9]. Higher preoperative PTH has been linked to more accurate MIBI and cytological predominance of individual tumors may also play a role in ability to detect their presence on MIBI [9].

This case adds to the literature by presenting a patient who initially had characteristics more commonly seen with a single PA but was ultimately diagnosed with 4-gland PH requiring reoperation. The patient initially had US findings typical of a single adenoma, which was identified and removed during surgery. Two additional hyperplastic glands were eventually identified on 4D-CT, which was not initially performed due to reported contrast allergy. The fourth gland appeared normal in size on 4D-CT but was determined to be hyperplastic intraoperatively. Interestingly, these additional enlarged glands were not visualized on initial US, repeat US, PET/CT, or MIBI. The patient ultimately required reoperation for removal of these additional hyperplastic glands. Had 4D-CT been performed at the time of initial diagnosis, multiglandular disease would have been detected, which would have prevented the need for repeat surgical procedure. Intraoperative PTH monitoring or further surgical exploration also could have led to earlier identification of multiglandular disease.

This case was also notable in that the multiple enlarged glands were unusually large to be seen in PH; typically, the enlarged glands in 4-gland hyperplasia are smaller than those seen in cases of single or double adenomas, but the largest gland in this patient weighed 3105 mg. Additionally, this patient's largest gland, which was not localized by US, MIBI, or PET/CT, was larger than any previous hyperplastic gland reported to be undetected on various imaging modalities. While it is not uncommon for parathyroid pathology to be undetected by localizing scans, this is typically with smaller adenomas and non-massive hyperplastic glands. No previous reported nonectopic hyperplastic glands of this size have been documented that were not detected on US, MIBI, or PET/CT. Intraoperative PTH testing, which unfortunately was not performed in this case, may also aid in risk stratification and diagnosis in patients with multiglandular disease.

Various gene mutations are associated with PH and PA. Somatic loss of one *MEN1* allele is seen in 25% to 40% of sporadic parathyroid adenomas, *cyclin D1* overexpression is observed in 20% to 40% of parathyroid adenomas and 31% of secondary hyperplastic glands, and *cyclin D1* gene translocation and oncogene action is present in 8% of adenomas [10]. Parafibromin testing of parathyroid tissue is also a useful tool in determining risk; negative stain is associated with malignancy, and with a higher risk of recurrence, metastasis, and mortality [11]. The patient presented here did not have any known pertinent family history and declined genetic testing. His parafibromin stain, however, was positive, which indicates a lower likelihood of CDC73 inactivating mutation [12].

## Learning Points

- Parathyroid hyperplasia may present atypically as a parathyroid adenoma on imaging.
- Hyperplastic parathyroid glands may be undetected by multiple imaging modalities, and hyperplasia of multiple glands cannot be ruled out by imaging alone.
- Intraoperative PTH measurements may aid in diagnosis and risk stratification.

## Contributors

All authors made individual contributions to authorship. G.D.R. was involved in the diagnosis and management of this patient and manuscript submission. S.N.H., M.B.G., and E.R. were involved in data compilation and manuscript preparation and editing. J.P.B. was involved in manuscript editing. All authors reviewed and approved the final draft.

## Data Availability

Original data generated and analyzed during this study are included in this published article.

## Abbreviations

4D: 4-dimensional
CT: computed tomography
DXA: dual-energy x-ray absorptiometry
MIBI: methoxyisobutylisonitrile (sestamibi)
PA: parathyroid adenoma
PET/CT: positron emission tomography/computed tomography
PH: parathyroid hyperplasia
PHPT: primary hyperparathyroidism
PTH: parathyroid hormone
US: ultrasound
